# Consumer Neuroscience: Attentional Preferences for Wine Labeling Reflected in the Posterior Contralateral Negativity

**DOI:** 10.3389/fpsyg.2021.688713

**Published:** 2021-10-14

**Authors:** Letizia Alvino, Efthymios Constantinides, Rob H. J. van der Lubbe

**Affiliations:** ^1^Center for Marketing and Supply Chain Management, Nyenrode Business University, Breukelen, Netherlands; ^2^Hightech Business and Entrepreneurship Group (HBE), Faculty of Behavior, Management and Social Sciences, University of Twente, Enschede, Netherlands; ^3^Cognition, Data, and Education, Faculty of Behavior, Management and Social Sciences, University of Twente, Enschede, Netherlands; ^4^Laboratory of Vision Science and Optometry, Faculty of Physics,Adam Mickiewicz University, Poznań﻿, Poland

**Keywords:** consumer neuroscience, neuromarketing, EEG, visuospatial attention, extrinsic cues, posterior contralateral negativity, N2pc, wine labeling

## Abstract

During the decision-making process, consumers notice, inspect, and visually scan different products. External characteristics of a product, such as design, packaging, label, and logo, have been shown to strongly influence how customers perceive, assess, and select a product. Marketers have put a lot of effort into determining the factors that trigger consumers’ visual attention toward products, using traditional research methods, self-reports, or observations. The use of neuroscientific tools to study consumer behavior may improve our understanding of how external characteristics influence consumers’ visual attention. Consumer neuroscience research shows that preferences for a product may already be reflected in brain activity before customers make a final decision. Using electroencephalography (EEG), we investigated whether the design of different wine labeling influences individual preferences, reflected in the neural activity related to visual attention. More specifically, we examined whether the posterior contralateral negativity (PCN) can be used to assess and predict consumers’ preferences for a specific product based on its external characteristics. The PCN is commonly used to estimate attentional selection by focusing on stimulus-side dependent EEG lateralization above parieto-occipital areas. We computed the PCN to assess whether a certain wine label caught participants’ visual attention and additionally by comparing the PCN with behavioral data (wine preferences and reaction times) to determine whether early effects of visual attention could predict participants’ final preferences for a specific label. Our findings indicate that the PCN provides relevant information on visual attention mechanisms for external characteristics, as the view of the four labels modulated PCN amplitude. We hope this study can help researchers and practitioners in examining the effects of external product characteristics on consumer choice by estimating the changes in the EEG that are related to visual attention.

## Introduction

A product’s external characteristics (or extrinsic cues) refer to any product features that can be manipulated without changing the essential attributes of the product (Olson and Jacoby, 1972; Veale and Quester, 2009; Jaafar et al., 2012; Yan et al., 2019). In marketing, external characteristics (e.g., brand, label, country of origin, and price) are often used to positively influence the consumer’s product quality perception (e.g., being cheap or expensive, hedonic or utilitarian, and safe or unsafe) (Veale and Quester, 2009; Abdullah et al., 2013; Spence, 2016; Ardeshiri and Rose, 2018; Yan et al., 2019). Studies suggest that consumer preferences for a product can be strongly influenced by its external characteristics (Pechmann and Ratneshwar, 1992; Lans et al., 2001; Lange et al., 2002; Bredahl, 2003; Veale and Quester, 2009). Researchers identified three conditions in which consumer preferences for a product are strongly affected by its external characteristics: (1) when consumers are not familiar with the product (2) when consumers do not have access to the internal attributes of the product, and (3) when consumers do not have enough knowledge to assess the quality of a product (Zeithaml, 1988; Underwood et al., 2001; Mueller and Szolnoki, 2010; Risius et al., 2019). Thus, the product external characteristics are very important in situations where the product is unknown to the consumer, or consuming the product is not possible before purchasing it and when assessing product quality is directly related to the consumers’ expertise about it. This condition is very common for beverage products, like wine. Research suggests that most wine consumers are forced to choose the wine based on its external characteristics (Tang et al., 2015).

Consumers might face one or more of the above-described situations while purchasing a bottle of wine: Consumers might not know the type of wine they are purchasing, they might be exposed to a large product assortment, and in most cases, it is not possible to taste the wine prior to purchase (Tang et al., 2015). They might also lack the knowledge on how to assess the quality of wine, even if they can taste it. Literature suggests that the label is among the most important external characteristics for wine choice (Thomas and Pickering, 2003; Goodman et al., 2005; Grunert, 2005; Cohen, 2009; Tang et al., 2014, 2015; Latiff et al., 2015). Wine labels are known to strongly influence consumer preferences and purchase decision-making (Thomas and Pickering, 2003; Grunert, 2005; Mueller and Lockshin, 2008; Orth and Malkewitz, 2008; Cohen, 2009; Latiff et al., 2015; Tang et al., 2015; Barwich, 2017). Gluckman (1986) identified that consumers perceive wine labels as one of their primary sources of information. Consumers rely on the label to collect important information about the wine, such as its country of origin, grape variety, year of production, and producer (Tootelian and Ross, 2000; Lange et al., 2002; Thomas and Pickering, 2003; Goodman et al., 2005; Hall and Mitchell, 2008; Tang et al., 2015).

Beyond the legal requirements that must be printed on a label for the product to be sold, the design of a label can suggest and communicate a lot of information about a wine. It can make the wine look expensive (even if it is not), appear fresh, and modern, or suggest a certain taste. In many cases, the label design and information provided offers reassurance that the wine will provide value for money in terms of performance and quality (Thomas and Pickering, 2003; Barber et al., 2006, 2007). Two important classifications of wine label designs are the “traditional” and the “modern/contemporary” labels (Batt and Dean, 2000; Boudreaux and Palmer, 2007; Hall and Mitchell, 2008; Elliot and Barth, 2012; Larson, 2012). Elliot and Barth (2012) noted that in the United States, modern, innovative, and distinctive labels are more attractive to younger consumers compared to the older consumers (who prefer more traditional styles). Other studies suggest that French consumers, whether young or old, novice or expert, still prefer wine with traditional labels in order to reduce perceived risk (Celhay and Trinquecoste, 2015).

Marketers often use techniques like observation, focus groups, and questionnaires to study consumers’ preferences for wine labels (Lange et al., 2002; Barber et al., 2006; Elliot and Barth, 2012; Celhay and Trinquecoste, 2015). However, traditional marketing techniques cannot always give an accurate and objective understanding of consumer behavior during wine selection (Ariely and Berns, 2010; Babiloni et al., 2014; Alvino et al., 2018, 2019b). In recent years, the rapid advance in neuroscience research has made it possible to use neuroscientific tools for business purposes. The use of neuroscientific techniques and tools for marketing purposes is known as Consumer Neuroscience or Neuromarketing.^1^ In our paper, the term Consumer Neuroscience is preferred. Consumer Neuroscience helps both researchers and practitioners to investigate how cognitive processes originate in the brain and identify the brain areas involved in the explication of cognitive functions underlying marketing-relevant behavior (Alvino et al., 2020). Consumer Neuroscience research addresses marketing-related issues such as advertising, branding, product experience, online experience, product development, and product pricing (Clement et al., 2017; Alvino et al., 2019a, 2020; Ma et al., 2019a,2019b; Sung et al., 2019; Ciceri et al., 2020; Fan et al., 2020; Hu et al., 2020; Yu et al., 2020; Liu et al., 2021).

Consumer Neuroscience can help companies to design and develop more successful and desired products by studying consumers’ physiological and neurophysiological responses to a product’s external characteristics, such as a label. Attention can be defined as the ability to focus on certain aspects of the environment while ignoring other information (Venkatraman et al., 2015). In particular, attention in the market field is the degree to which consumers focus on a stimulus, a prerequisite for information processing and, therefore, a key step in the consumer’s decision-making process (Varela et al., 2014; Krucien et al., 2017; García-Madariaga et al., 2019). As a consumer’s attention toward a product is reflected in the neural processing of visual stimuli (Stasi et al., 2018; Alvino et al., 2019b; Karmarkar and Plassmann, 2019), studying the allocation of visual attention might help to define how a product’s external characteristics are processed in the brain. According to Clement et al., 2017), visual attention is a key component in consumers’ decision-making process since information must be visually noticed to influence choice. Similarly, studies suggest that product preferences at least partially depend on the amount of attention that they receive during the decision-making process (Krajbich et al., 2010; Glimcher and Fehr, 2013; Chen et al., 2019). Consumer Neuroscience studies have investigated changes in consumer’s visual attention mechanisms related to different label characteristics (e.g., attractive vs. unattractive and presence of sustainability information), type of ingredients used (earthworm flowers vs. grain crackers), visual elements of the wine labeling (e.g., text vs. images and different design), and consumer knowledge about wine (non-expert and expert wine drinkers; Russo, 2015; van Loo et al., 2015; Laeng et al., 2016; Khachatryan et al., 2017; Russo et al., 2020). Except for the study of Russo et al. (2020), all these studies were conducted using eye tracking (ET) as the main research method.

Eye tracking has been widely used in consumer neuroscience research to study visual behavior (e.g., point of fixation, gaze, and pupil dilatation), customers’ visual attention mechanisms, and consumers’ engagement (Alvino et al., 2020). However, the literature suggests that the measurement of eye movements is not always sufficient to understand how consumers focus their attention, for instance why a label catches consumers’ attention. In fact, eye movements are discrete events (limited to visual or written language comprehension). Several authors suggest that eye movements are partly dependent on higher cognitive processes (Rayner, 2009; Luck and Kappenman, 2011; Rayner et al., 2015; Rayner and Reingold, 2015; Luke et al., 2018). This makes eye movements also relatively slow compared to other mechanisms (e.g., brain activity) (Luck and Kappenman, 2011). For instance, when reading text, eye movements are influenced on a moment-by-moment basis by a variety of linguistic factors, such as word frequency, predictability, and syntactic complexity (Rayner, 1998, 2009; Rayner and Reingold, 2015; Luke et al., 2018). Thus, eye movements are often a consequence of cognitive processes that may already be reflected in ongoing brain activity before the actual eye movement is executed.

As most cognitive processes occur within tens to hundreds of milliseconds (Freeman and Quiroga, 2012; Cohen, 2014), it is possible that consumer neuroscience tools that identify and analyze brain activity are more effective in studying the visual allocation of attention mechanisms and individual preferences. Literature suggests that electroencephalography (EEG) is a suitable tool to measure visual attention mechanisms. EEG is a non-invasive brain imaging method that detects brain electrical activity using different electrodes placed on the scalp (Abhang et al., 2016; Alix et al., 2017). EEG has an excellent temporal resolution (Burle et al., 2015; Bilucaglia et al., 2020); thus, it can capture the dynamics of brain processes in the time frame in which they occur (Freeman and Quiroga, 2012; Cohen, 2014). EEG is well suited to capturing the fast, dynamic, time sequenced cognitive events underlying the visual allocation of attention (Cohen, 2014). This permits the identification, within a functional time window, of neurophysiological correlates of the exposure to marketing stimuli, such as external cues (e.g., labels, packaging design) (Bazzani et al., 2020). Studies show that changes in electrophysiological measures can be useful for examining the perceptual and cognitive processes that occur in response to marketing stimuli (Ma et al., 2019a,b).

To study whether individual preferences for different wine labeling are related to the allocation of visual attention, we focused on changes in the posterior contralateral negativity (PCN). Parameters of the PCN^2^ are analyzed in order to assess whether a certain bottle/label caught visual participants’ attention. The PCN is “*an established electrophysiological marker for examining (millisecond-by-millisecond) the deployment of focal attention in visual space*” (Töllner et al., 2012; pp 1556). Parameters of the PCN reflect the dynamics of visuospatial attention processes and provide a reliable and valid temporal measure of target localization (Geyer et al., 2010; Töllner et al., 2011; Vossel et al., 2014). The PCN expresses an increased negativity above visual brain areas (posterior electrodes) contralateral to the stimulus position in a time window of approximately 175 and 300ms (or even less) after the stimulus presentation. This parameter can be used as a marker that traces the transition from when the stimulus (e.g., a label) reaches a receptor (e.g., retinal cell) to the focal attentional stage to target selection, thus when the stimulus is perceived and successively selected (Töllner et al., 2011). Numerous psychological and neuroscientific studies used the PCN in order to examine how the timing and the allocation of visuospatial attention is modulated by stimulus intensity, stimulus saliency, aging, and set size (Van der Lubbe et al., 2001; Van der Lubbe and Verleger, 2002; Geyer et al., 2010; Töllner et al., 2011; Vossel et al., 2014).

Based on the aforementioned considerations, our study aims to *investigate whether changes in the brain activity and individual preferences for wine labeling*^3^ *is related to the allocation of visual attention*. To achieve our aim, we carried out a laboratory experiment using EEG. During the experiment, thirty-one volunteers were exposed to four different examples of wine labeling, in pairs of two, which were presented on the left and right side of a computer screen. We used a within-subjects design and carried out the experiment in two sessions. Participants were asked to select the preferred wine labeling (by pressing a button on the corresponding side) while their electrical brain activity was recorded.

The current study adds to previous research by investigating whether the PCN can be used to assess and predict consumers’ preferences for wine labels.

## Methodology

### Participants

Before the experiment, participants were asked to fill in a questionnaire about their drinking habits, their wine knowledge, and the Alcohol Use Disorders Identification Test (AUDIT). Both questionnaires and the AUDIT test were sent by email. We used the AUDIT to assess whether volunteers could participate in our experiment. This test identifies participants who display hazardous (or risky) drinking behavior, harmful drinking, or alcohol dependence. Participants with a score higher than 19 in the AUDIT were excluded as they can be considered to abuse alcohol, which may lead to deviant results (Lacoste-Badie et al., 2020). Two subjects were not invited to join the experiment as they score was higher than 19. All selected participants had a score lower than 18.

EEG was recorded from thirty-one participants. The participants were all volunteers, so they did not receive an incentive to participate in this study. Participants were mostly students and/or employees of the University of Twente (The Netherlands). The study was evaluated by the ethical committee of the BMS faculty and was carried out in line with the declaration of Helsinki.

When participants arrived at the laboratory, they were also tested for handedness and color blindness. We used Annett’s Handedness Inventory (Annett, 1970) to test handedness as this is an important factor in the investigation of brain lateralization (van Strien, 1992, 2003). The handedness test revealed that twenty-eight participants were right-handed, and three participants were left-handed. Several studies suggest that color vision deficiency (CVD) is one of the most common types of vision deficiency (Ekhlasi et al., 2021). In this experiment, participants were asked to assess the preferences for wine labeling with different designs and colors. We tested participants’ possible defects of color vision using the Ishihara test (Birch, 1997). The test consists of a number of colored plates, namely, Ishihara plates, each of which contains a circle of dots appearing randomized in color and size (Birch, 1997; Ekhlasi et al., 2021). Participants were asked to report the colored numbers in the figures. All participants had normal color vision.

Six participants were excluded from the original sample for different reasons. For two participants, a different amplifier was used in the second as compared to the first session, due to EEG equipment failure. Two participants were not able to take part in the second session. Two other participants were excluded because of excessive artifacts in their EEG recordings. The final sample consisted of twenty-five participants between 18 and 40years of age (*M*_age_=26.4, *SD*=4). In total, ten participants were female (*M*_age_=28, *SD*=5, ranging from 23 to 39years) and fifteen participants were male (*M*_age_=25.5, *SD*=3.1, ranging from 19 to 31years). All volunteers had no history of neurological illness or damage, were not using drugs or psychiatric medication, and had normal or corrected-to-normal vision and no color blindness. Participants’ knowledge of wine was based on self-report. Only participants with no knowledge or little knowledge of wine were selected to take part (Bruwer and Li, 2007; Laeng et al., 2016). Based on the questionnaire’s results, eleven participants could be considered as inexperienced, while fourteen participants displayed basic knowledge. Participants had no prior knowledge of the wines presented during the experiment.

### Procedures and Task

When participants arrived at the laboratory, they were asked to sign an informed consent form. Participants received detailed written and verbal instructions on all the tasks they were going to perform in the experiment. The volunteers were invited to sit in a comfortable chair, and EEG electrodes were applied. The room was sound-attenuated and illuminated. Participants were placed at a distance of approximately 100 (cm) at the eye level in front of a 24-inch AOC G2460P LED computer screen. Participants were asked to relax and to reduce sudden movements and blinking in order to prevent distortion of the EEG signal. An experimenter sat nearby throughout the experiment to check the procedure and to answer any questions.

The experimental design was set up considering repeated measurements (within-subject design). In a within-subject design experiment, each individual is exposed to more than one of the treatments being tested, whether it be playing a game with two different parameter values, being treated and untreated or performing tasks under more than one external stimulus (Charness et al., 2012). These experiments are more naturally aligned with most theoretical mindsets. For instance, a theorist is likely to imagine an agent in a market reacting to a price change, not two agents in separate markets with different prices (Charness et al., 2012). In this experiment, participants took part in two sessions (*Session 1* and *Session 2*), which were separated by 2weeks. A time frame of 2weeks was chosen in order to reduce the possibility that volunteers would remember the preferences indicated for each wine but at the same time examine the consistency of their preferences.

The study employed a “Stimulus Discrimination” task that required a right-hand or left-hand button press in response to the presentation of a set of two wine bottles/labels. Participants were instructed to press the key on the side that corresponded with the label/bottle they preferred. Responses were made on a standard QWERTY keyboard, with the left index finger positioned on the “left Ctrl” key, and the right index finger on the “right Ctrl” key. Stimulus presentation was controlled by Presentation software (Neurobehavioral Systems, Inc., 2012).

**Figure 1:**
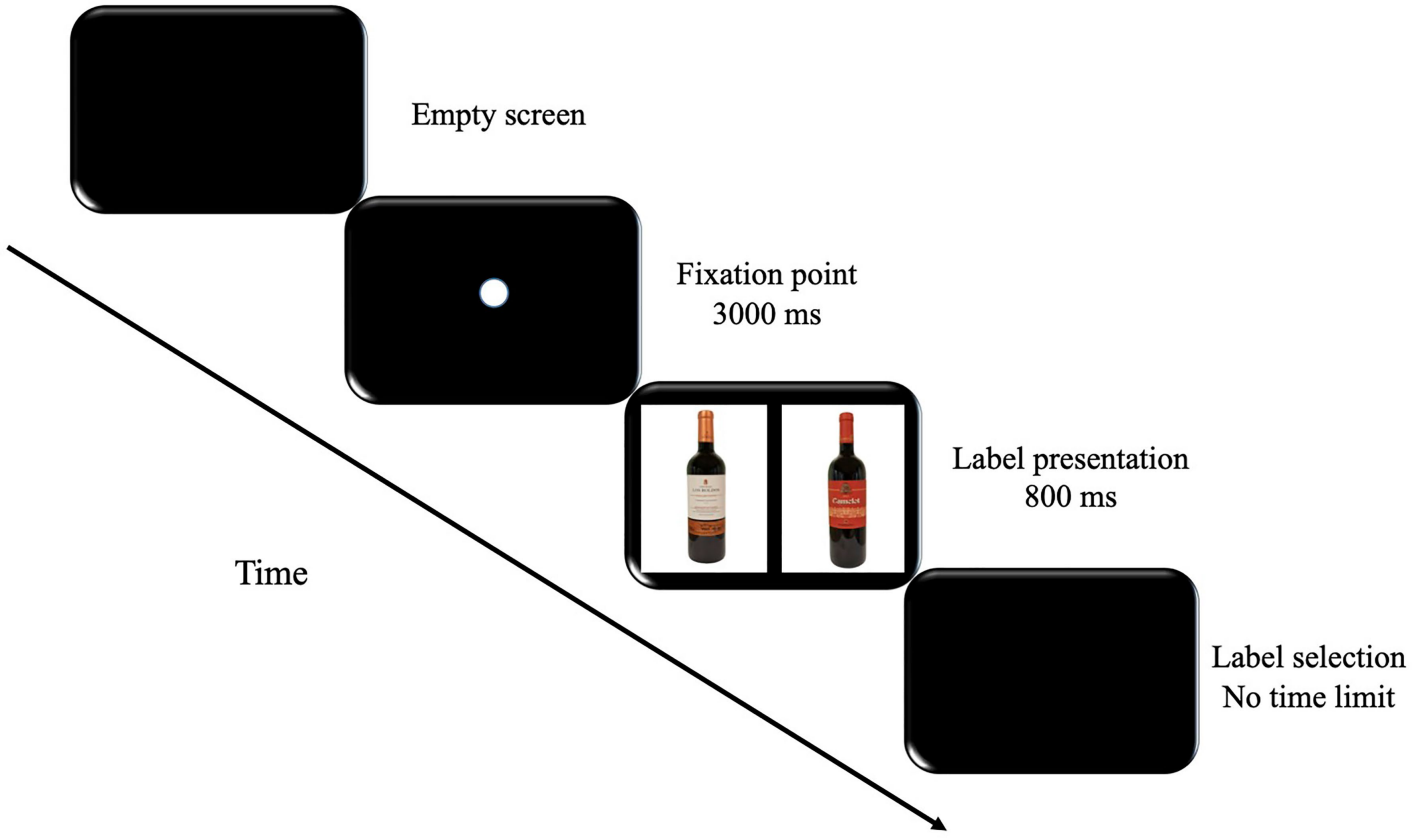
Experimental procedure. Participants were asked to choose the preferred wine after viewing the labeling presentation.

Each participant completed ten practice trials to get familiar with the task before the real experimental phase. As shown in Figure 1, each trial began with a white fixation point appearing in the center of the computer screen, followed by an interval of 3000ms before the white fixation point turned red (for 200ms). Then, a pair of bottles/labels was presented on the screen for 800ms and the participants could choose the preferred bottle/label. Participants could freely decide (1) when to press one of the two buttons (no time limit) and (2) whether they wanted to press the right or the left button.

The stimulus discrimination task was divided into four blocks, and the participants had 1min of rest at the end of each block. The blocks contained 96 stimuli each. Overall, the participants saw a succession of 384 sets of pictures of four different examples of wine labeling. The average duration of the task was between 42 and 47min, including a time break between the blocks.

### Stimuli

The pictures of four examples of wine labeling were used as stimuli. The wines were selected based on the type of grape, price, country of origin, and label. The wines selected had the same type of grape (Cabernet Sauvignon, 100 percent) but a different country of origin (Chili and Italy). The wines were in a low-to-moderate price range (€3–€27; Boudreaux and Palmer, 2007). Two wines were moderate-priced wines (price category: €24–€27), while the two other wines were low-priced wines (price category: €3–€5). The prices were compared on the same website to have a realistic evaluation of the wines. All the wines can easily be bought online. For simplicity, we divide the wines into Cheap (C: €3–€5) and Expensive (E: €24–€27).

**Figure 2:**
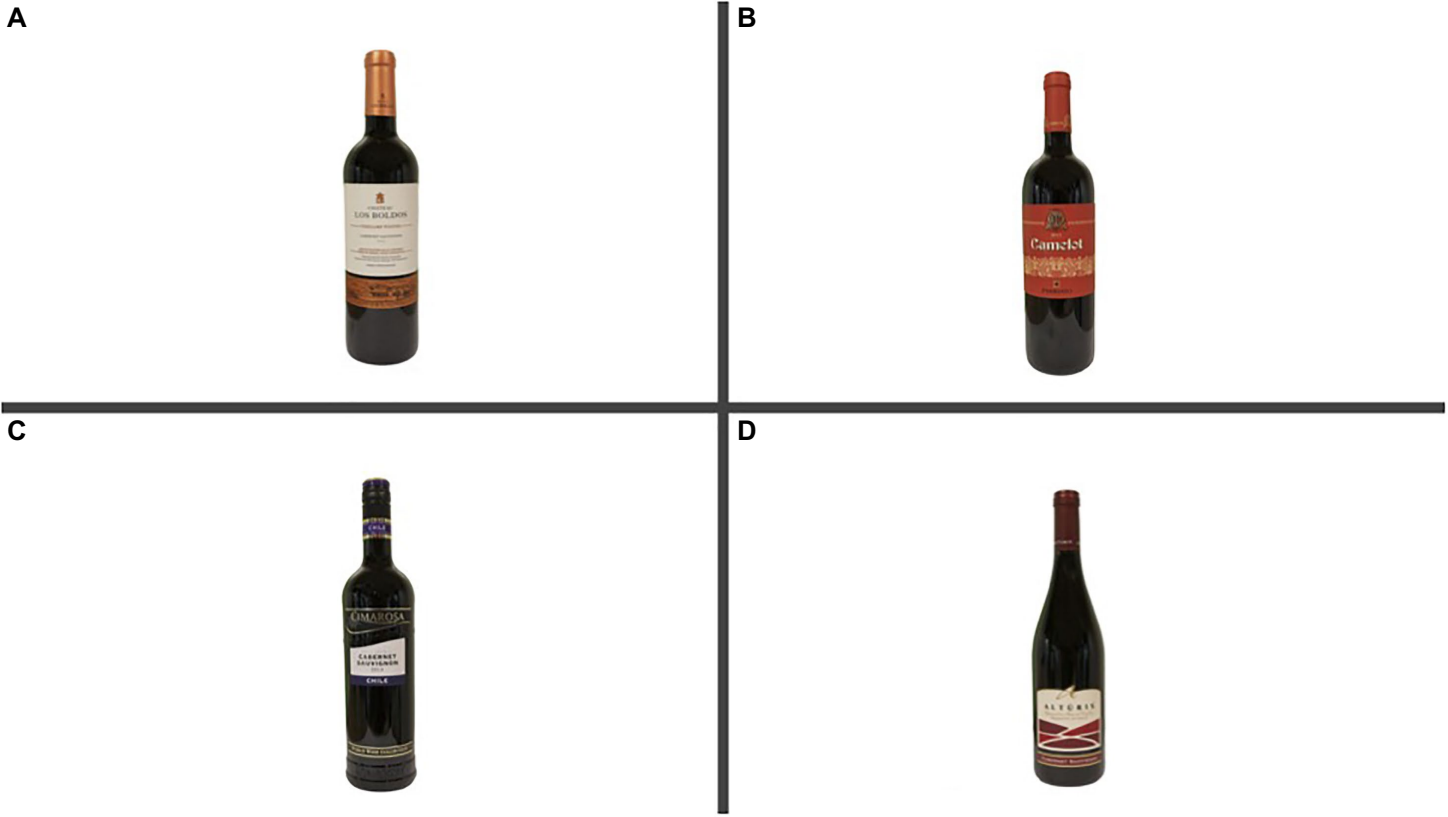
Experimental stimuli (Wine bottles and labels). Chilian Expensive **(A)**, Italian Expensive **(B)**, Chilean Cheap **(C)**, and Italian Cheap **(D)**.

Marketing literature suggests that wine label design could be classified as either “traditional” or “modern/contemporary” (Batt and Dean, 2000; Boudreaux and Palmer, 2007; Hall and Mitchell, 2008; Elliot and Barth, 2012; Larson, 2012). Based on previous studies, the labels were selected according to specific patterns hue and color (dark or bright), images (e.g., chateaux or animal), different writings (white, black, or gold), bottle shape (standard shape or odd shape), and overall design (simple or complex; Batt and Dean, 2000; Boudreaux and Palmer, 2007; Elliot and Barth, 2012; Sáenz-Navajas et al., 2013). As shown in Figure 2, the two Chilean wines had a simple and traditional label, and for both wines, the type of wine and the production year was clearly written in the middle of the label. In particular, the label of the Chilean Expensive (CE) wine was white and bronzed, with a vineyard drawn at the bottom of the label. The country of origin was written in small characters. The Chilean Cheap (CC) label was white with blue sides; the name of the wine was written in gold characters, and the bottle had a plastic cork. Both the production year and country of origin were visible on the label. Overall, the two labels had an old heritage style (Elliot and Barth, 2012). The label of the two Italian wines had a more modern design. Both labels had contemporary fonts, abstract forms, bright color (red), and asymmetrical shapes. The label of the Italian Expensive (IE) wine was red with gold and white characters. The production year, the names of both wine and producer were clearly visible. The label had a golden drown of warriors to revoke the name of the wine. The label did not show the country of origin. The Italian Cheap (IC) label was white and red, and small red patterns were presented. However, the bottle had a peculiar shape, different from the other wines (Boudreaux and Palmer, 2007; Elliot and Barth, 2012). The country of origin, type of wine (Cabernet Sauvignon) was also visible (Elliot and Barth, 2012).

The wine bottles were photographed using a NIKON D3300 camera. The bottles were positioned on a white backdrop, and they were illuminated with different daylight bulbs to balance the pictures (the labels were comparable in size and luminance). Adobe Photoshop CC (2015) software was used to erase the background and regulate the size and luminance. The four pictures were stored as 300-pixel JPEG files. Images were displayed aligned vertically in the center of the screen (as shown in Figure 1). Finally, the stimuli were digitally presented on a 24-inch monitor.

### Behavioral and EEG Data Acquisition and Analysis

The EEG was recorded continuously from 32 active Ag/AgCl electrode (wet electrodes) sites using an EasyCap-62 channel cap (standard international 10–20 system layout) connected to an ActiChamp amplifier, with BrainVision Recorder software (version 1.21.0102). The electrodes were located at the following sites: AFz, AF3, AF4, AF7, AF8, F1, F2, F5, F6, FCz, FC3, FC4, FT7, FT8, C3, C4, C5, C6, CPz, CP3, CP4, TP7, TP8, P1, P2, P5, P6, POz, PO3, PO4, PO7, and PO8. The horizontal and vertical electro-oculogram (hEOG and vEOG) were also recorded. Two electrodes were placed at the side of both eyes to measure the electrical activity generated by horizontal eye movements. Electrodes located on the infraorbital and supraorbital regions of the left eye placed in line with the pupil enabled to measure vertical eye movements and blinks. The resistance of the electrodes was kept below 10 kΩ by using electrode gel and standard procedures to improve conductivity.

EEG data were analyzed with BrainVision Analyzer v. 2.1.1 software. The continuous data were epoched from 500ms prior up to 2500ms after presenting the stimuli (wine bottles/labels). An initial baseline was set from −100 to 0ms before presenting the stimuli. EEG was corrected for eye movement-related artifacts *via* artifact rejection and Ocular Correction ICA (Independent Component Analysis). Trials with amplitude differences exceeding ±50μV on the hEOG channel and±100μV on the vEOG channel were marked to remove segments with horizontal eye movements and eye blinks from 200ms before until 200ms after presenting the stimuli. This procedure left on average 79.5% (*SD*=15.8%) of the presented trials per participant. After the Ocular Correction, another artifact rejection was applied (criteria were set to remove trials with differences of more than ±150μV, a gradient criterion of 50μV/ms, and a low activity criterion of 0.1μV for 50ms). EEG channels with artifacts were marked and were not included in the data analysis. Finally, lateralized EEG potentials as a function of the to-be- attended side (details below) were determined for all homologue electrode pairs and labels.

During the experiment, two wines were presented simultaneously on the computer screen, one on the left and the other on the right side of a computer screen. To precisely trace the allocation of focal attention, we determined the PCN. Increased negativity above visual brain areas contralateral to the relevant visual stimulus (here, the relevant wine labeling) was expressed by detecting potential changes in the PCN (Jolicoeur et al., 2008; Vossel et al., 2014). The presence of a PCN for a specific labeling points to an attentional preference for this labeling, and the more negative the PCN, the more we assumed this labeling seems to be preferred. The amplitudes of the PCN were assessed within 40-ms time windows from 0ms to 280ms poststimulus at the components’ most typical electrode sites PO7/8, by determining lateralized potentials. Here is an example of the procedure of computing the PCN for Label 1: {[PO8-PO7 (Label 1 left visual field (LVF) - Label 2/3/4 right visual field (RVF)]+[PO7-PO8 (Label 2/3/4 LVF - Label 1 RVF)]}/2. Thus, the PCN for each label was computed by comparing it with all other labels on the ipsilateral side. This procedure was used for all labels and for each session separately. We applied the same procedure for all symmetrical electrode sites (e.g., C3 and C4) to be able to create topographical maps displaying event-related lateralizations (ERLs; e.g., see Van der Lubbe et al., 2001). According to Töllner et al. (2011) and Töllner et al. (2012) the PCN is present from 175 to 300ms after stimulus onset. In our case, we decided to export the data from 0 to 280ms in seven time windows of 40ms each. PCN values for all time windows were used for the statistical analysis. These data were analyzed with SPSS (IBM SPSS V25.0).

For the behavioral data, participant’s preferences for wine labeling and reaction time (RT) were recorded during the experiment. The participant could select their preferred wine labeling by using the left or right Ctrl key of the keyboard (depending on where the preferred label would appear). Thus, labeling preferences were determined based on the total number of trials in which they selected a specific wine. The reaction times were not analyzed as there was no real-time limit to choose the preferred wine.

Reaction time indicates the time a participant takes to respond to a task (Ramsøy, 2014). Reaction times measure individuals spontaneous or “gut instinct” responses to a stimulus (Calvert et al., 2019). In this study, we measured the amount of time it took to participants to select the desired wine labeling. RT was also determined based on the total amount of trials in which they selected a specific label.

A repeated measures ANOVA was used to analyze both the EEG, the consumer preferences and reaction time. For the behavioral data, the repeated measures ANOVA was used to analyze differences in the participants’ responses to each wine labeling (*Labeling Preferences*). As mentioned before, this was determined based on the number of times a label would be selected during each session. Participants’ responses were analyzed with the factors Labeling (*CE, IE, CC, and IC*), Session (*Session 1 or Session 2*), and Side (left or right). Then, differences in electrophysiological measures (changes in PCN values) were analyzed for the four bottles/labels, the sessions, and the different time windows (*40ms each*). For the analysis, seven different time windows (from 0 to 280ms) were selected. For both EEG and behavioral data, associated Degrees of freedom, *F*-values, *value of p*s, Means, and Std. Deviation were reported. The associated Mauchly’s test of sphericity was also analyzed. Corrected results (Greenhouse–Geisser or Huynh-Feldt correction)^4^ were reported when the assumption of sphericity was violated.

## Results

### Behavioral Data

The results show that there was no significant effect of Side of stimulus presentation [*F*_(1,24)_=0.14; *p*=0.707] and Session [*F*_(1,24)_=2.39; *p*=0.136] on participants preferences for specific wine bottles/labels. Thus, participants’ preferences were not affected by the session and the presentation side of the bottle/label. However, results show that there was an effect of Labeling on participants’ responses [*F*_(3,22)_=12.29; *p*<0.001].

**Figure 3:**
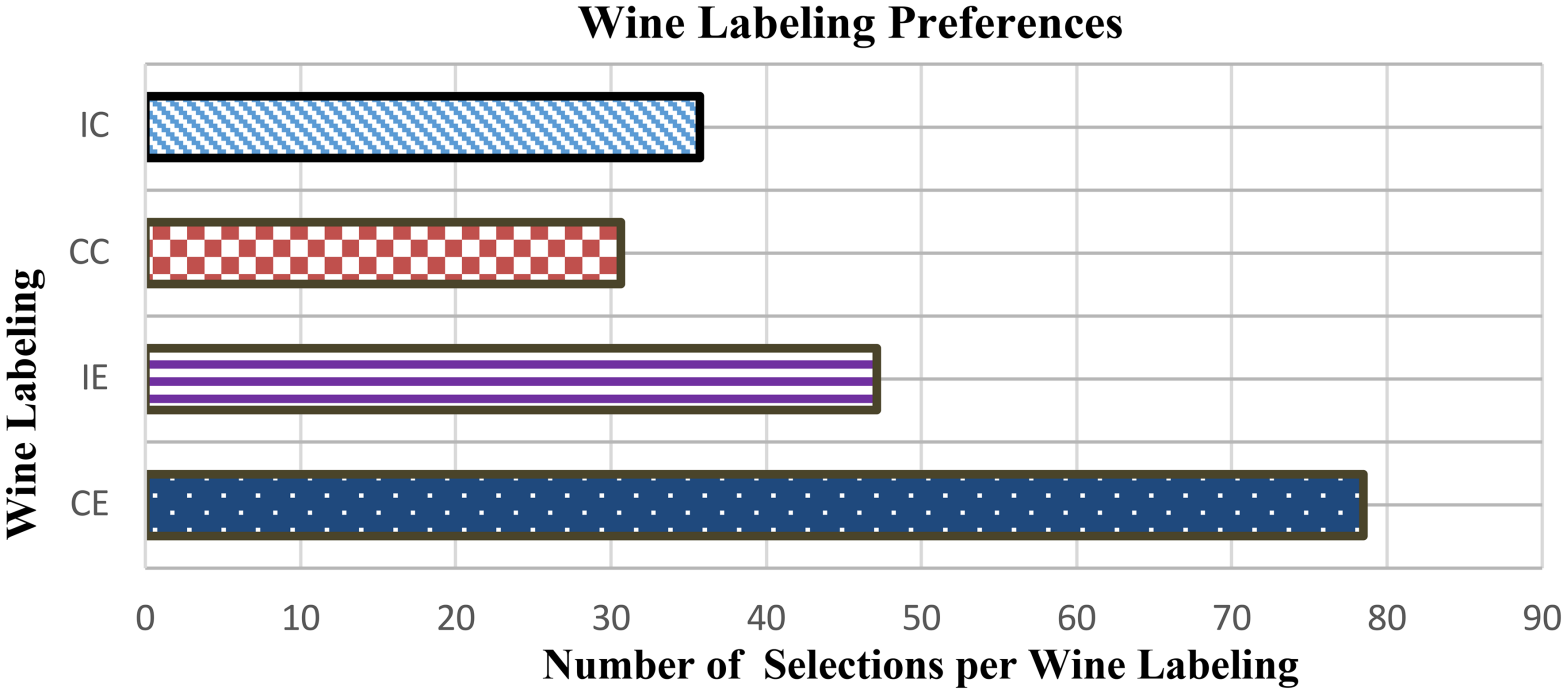
The figure shows the participants’ preferences for wine labeling (number of times the participants selected the wines).

Separate comparisons between the different wine labeling were performed to determine the participants’ preferences. There was a preference for the Chilean Expensive (CE) as compared to the other wine labeling (see Figure 3). Specifically, there was a significant difference in the number of times that the CE was selected as compared to the Italian expensive (IE) [*t*_(24)_=7.560; *p<* 0.0001], Chilean cheap (CC) [*t*_(24)_=9.642; *p*<0.0001] and Italian cheap (IC) wine [*t*_(24)_=10.268; *p*<0.0001]. Similarly, there was a strong effect of IE on participant’s preferences compared to CC [*t*_(24)_=3.333; *p*<0.001]. A slight difference was observed IE compared to IC [*t*_(24)_=2.533; *p*=0.013]. However, no significant differences were found between CC and IC [*t*_(24)_=−1.057; *p*=0.293]. As shown in Figure 3, the most preferred wine labeling was CE, followed by IE, IC and CC.

A repeated measures ANOVA was also conducted on RT. The results show that there was no significant effect of session [*F*_(1,24)_=0.38; *p*=0.53] on participants’ reaction times for specific wine labeling. Thus, participants’ reaction times were not affected by the session. However, the main effect of the labeling was slightly significant [*F*_(3,22)_=5.85; *p*=0.008]. This means that there was a difference in the response time of participants for the wine labeling. Overall, participants were more likely to make quicker decision if they were selecting the preferred bottle/label. As shown in Table 1, reaction times for CE were faster than for the other labeling (IE, CC, and IC). Participants took more time to select the least preferred labeling (CC).

**Table 1:** Mean of the participants’ reaction times for each example of wine labeling, and standard error of the mean.

Label	Mean	Std. Error
CE	762.402	41.275
IE	860.673	47.112
CC	956.514	68.976
IC	886.368	43.461

### EEG Data

To verify whether there were changes in the PCN amplitudes as a function of Labeling (*CE, IE, CC, and IC*), Time window (7), and Session (2), a repeated measures ANOVA was performed.

**Figure 4:**
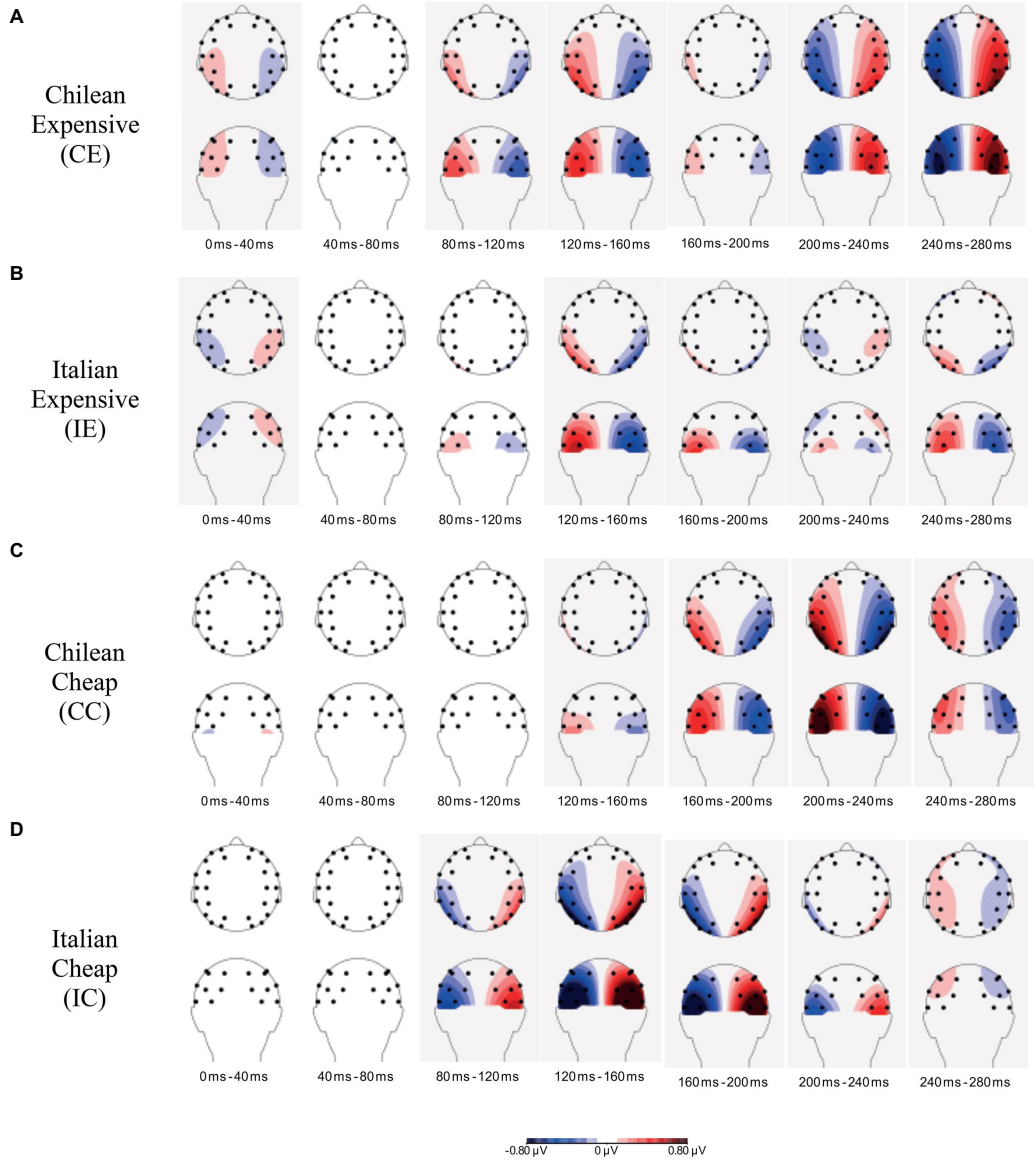
Topographical maps determined for 40-ms time windows (from 0 until 280ms) after stimuli presentation for all four wines: Chilean Expensive **(A)**, Italian Expensive **(B)**, hilean Cheap **(C)** and Italian Cheap **(D)**. Contra-ipsilateral differences relative to the label of interest are projected on the left hemisphere, while ipsi-contralateral differences are projected on the right hemisphere.

The results revealed no differences in the PCN amplitudes between the two sessions [*F*_(1,24)_=1.27; *p*=0.271] and also no effect of Time Window [*F*_(3.128,75.072)_=1.47; *p*=0.230], but a major effect of Labeling (*F*_(2.039,48.943)_=11.11; *p*<0.0001). An interaction was additionally observed between Time Window and Labeling [*F*_(5.887,141,296)_=13.5; *p*<0.0001], but not between Time Window, Labeling, and Session [*F*_(8.33,199.91)_=1.4; *p*=0.204]. The results suggest that there was a change in participants’ brain activity over time that differed between the wine labeling (*IE, CE, IC, and CC*). These changes over time are also clearly visible in Figure 4.

In order to analyze changes in participants’ brain activity over time as a function of Labeling, separate analyses were performed for the seven time windows. As this implies testing ten separate contrasts for each time window, we employed a critical value of p of 0.005 (i.e., we applied Bonferroni correction). For both the first (0 to 40ms) and second (40 to 80ms) time windows, results show no effects of Labeling (*p*>0.394) on PCN amplitudes.

In the third time window (80 to 120ms), an effect of Labeling was observed [*F*_(3,72)_=8.7; *p<* 0.0001]. Table 2 shows the mean of the PCN amplitudes for the four labels in the two sessions. Contrast analyses for the third time window revealed major differences between the CE and the IC wine [*F*_(1,24)_=17.7; *p*<0.0004]. The critical value of p was not crossed for the other contrasts. The mean amplitudes in Table 2 show a clear PCN for the IC wine, while an opposite effect is present for the CE wine, suggesting that within this time window participants attend to the IC and not to the CE label.

**Table 2:** Mean of the PCN amplitudes for each bottle/label averaged across sessions from 80–120 ms, standard errors of the mean, and results of *F* tests as deviation from “0.”

Wine Label	Mean	Std. Error	*F*	*P*
CE	0.347	0.104	11.2	0.003^*^
IE	0.168	0.081	4.3	0.05
CC	−0.033	0.094	0.1	0.725
IC	−0.478	0.132	13.1	0.001^*^

* *This value of p can be considered significant as it is below the critical value of 0.005*.

**Table 3:** Mean of the PCN amplitudes for each bottle/label averaged across sessions from 120 to 160ms, standard errors of the mean, and results of *F* tests as deviation from “0.”

Wine Label	Mean	Std. Error	*F*	*P*
CE	0.435	0.118	13.7	0.001^*^
IE	0.578	0.162	12.8	0.002^*^
CC	0.210	0.152	1.9	0.181
IC	−1.199	0.177	45.7	< 0.0001^*^

* *This value of p can be considered significant as it is below the critical value of 0.005*.

In the fourth time window (from 120ms to 160ms), a main effect of Labeling on PCN amplitude was observed [*F*_(1.982,47.560)_=21.194; *p*<0.000001]. Contrast analyses revealed major differences between the IC wine as compared to the CE, IE, and CC wine [*F*_(1,24)_>24.354; *p*<0.0001]. Inspection of the mean amplitudes in Table 4 again shows a clear PCN for the IC wine, while opposite effects seem present for the CE and IE labeling, suggesting that participants paid attention to the IC wine and did not attend to the CE and IE wines.

**Table 4:** Mean of the PCN amplitudes for each bottle/label averaged across sessions from 160 to 200ms, standard errors of the mean, and results of *F* tests as deviation from “0.”

Wine Label	Mean	Std. Error	*F*	*P*
CE	0.076	0.091	0.7	0.41
IE	0.421	0.130	10.5	0.003^*^
CC	0.501	0.094	28.3	< 0.0001^*^
IC	−0.984	0.132	55.2	< 0.0001^*^

* *This value of p can be considered significant as it is below the critical value of 0.005*.

In the sixth time window (from 200 to 240ms), again a main effect of Labeling on PCN amplitudes was found [*F*_(3,75)_=14.4; *p*<0.000001]. In this case, contrast analyses revealed no differences between the CE and IC label [*F*_(1,24)_=0.696; *p*=0.413], but differences were observed between the CC and IC label (*F*_(1,24)_=25.043; *p*<0.0001) and between the CC and CE label [*F*_(1,24)_=26.993; *p*<0.0001]. The mean amplitudes for this time window for each wine are presented in Table 5. It seems that now participants were paying attention to both the CE and IC wines, while they were clearly not paying attention to the CC wine.

**Table 5:** Mean of the PCN amplitudes for each bottle/label averaged across sessions from 200 to 240ms, standard errors of the mean, and results of F tests as deviation from “0.”

Wine Label	Mean	Std. Error	F	P
CE	−0.621	0.171	13.3	0.001^*^
IE	0.230	0.126	3.3	0.081
CC	0.808	0.135	35.8	< 0.0001^*^
IC	−0.413	0.155	7.1	0.014

* *This value of p can be considered significant as it is below the critical value of 0.005*.

In the seventh time window (from 240 to 280ms), a main effect of Labeling on PCN amplitude was observed [*F*_(2.391,57.394)_=5.948; *p*=0.002737]. Contrast analyses revealed differences between the CE label and the IE and CC labels [*F*_(1,24)_>10.729; *p*<0.0032], but for the other contrasts, the critical value of p was not crossed. Inspection of the mean amplitudes for this time window (Table 6) suggests that the participants were no longer paying attention the IC wine, but attended to the CE wine.

**Table 6:** Mean of the PCN amplitudes for each bottle/label averaged across sessions from 240 to 280ms, standard errors of the mean, and results of F tests as deviation from “0.”

Wine Label	Mean	Std. Error	F	P
CE	−0.798	0.243	10.8	0.003^*^
IE	0.459	0.197	5.5	0.028
CC	0.264	0.126	4.4	0.047
IC	0.072	0.204	0.1	0.726

* *This value of p can be considered significant as it is below the critical value of 0.005*.

## Discussion

Investigating consumer behavior with products helps companies to understand how consumers select a product and what role external characteristics (e.g., label and price) play an important role in product differentiation. In marketing research, a lot of attention has been devoted to study consumer preferences for product external characteristics (Olson and Jacoby, 1972; Inscha and McBride, 2004; Aaker et al., 2011; Armstrong et al., 2014). Despite decades of scientific effort, much is still unknown about the effects of product external characteristics on consumer decisions and preferences.

Consumer neuroscience studies show that our preferences for a product may already be reflected in our brain activity long before we make a final decision (Plassmann et al., 2012; Ma et al., 2019a,b; Sung et al., 2019; Alvino et al., 2020; Ciceri et al., 2020; Fan et al., 2020; Yu et al., 2020). The use of neuroscientific tools to study consumer behavior could improve our understanding of how external characteristics influence consumers’ preferences for a product (Plassmann et al., 2012; Alvino et al., 2020). In particular, consumer neuroscience tools can be used to examine the psychological and neural mechanisms that underlie visual attention (Russo, 2015; van Loo et al., 2015; Laeng et al., 2016; Khachatryan et al., 2017; Russo et al., 2020).

The aim of this study was to investigate whether preferences for specific wine labeling are also reflected in brain measures derived from the EEG. Here, we specifically focused on the amplitude of the PCN with the idea that the labeling that eventually is mostly preferred will also induce a larger PCN than the other labels. The PCN (approx. 175 to 300ms after stimulus implementation) can provide information about the role of visual attentional selection for the final decision. For instance, the presence of a PCN may imply that a participant is already attracted by the preferred label even before selecting it, or vice versa a label might initially attract attention (due to certain characteristics) but not be the preferred/selected label. Similarly, a label does not directly attract attention but may in the end be the most preferred label, suggesting that additional aspects apart from attention-attracting features play an important role.

The findings of our study (electrophysiological data) show that different label characteristics have an influence on brain activity. The results clearly reveal that the amplitude of the PCN differed between the employed wine labeling. The data suggest the presence of a PCN for two of the presented labels (CE and IC), while opposite effects seem present for the IE and CC labels, suggesting reduced attention for these labels. These changes are also visible over time. The earliest sign of the PCN was present for the IC label within the 80- to 120-ms time window, which remained until the 200- to 240-ms time window. A relevant observation of this early PCN is that it remains more or less restricted to posterior brain areas, suggesting that it reflects attentional selection and not hand motor activation, which can be observed above central areas (i.e., as measured at the C3 and C4 electrode locations). In fact, the behavioral data indicate that the IC label is only the third preferred label. A possible explanation is that the IC label had a salient and visually appealing aspects embodied in the label (contemporary fonts and abstract forms) and the bottle had a peculiar shape, different from the other wines (e.g., elongated neck; Elliot and Barth, 2012). Our interpretation of these results is that the bottle shape may have attracted participants’ attention, but in the end did not lead to a preference for this label. For the CE label, the earliest sign of the PCN is within the 200- to 240-ms time window, which further increased in the 240- to 280-ms time window. Importantly, in this case the topography of the PCN seemed to spread more to hand motor areas, suggesting not only that this label was paid attention, but also preferred as it may have led to hand motor activation. This might mean that the observed lateralization is actually a combination of the PCN with the lateralized readiness potential (LRP, e.g., see Van der Lubbe et al., 2001). As the behavioral data also point out in the same direction (both preferences and RT), our assumption is that participants indeed seemed to prefer the CE label.

The behavioral data show participants’ preferences for the four labels. Participants favored the labels of the more expensive wines (CE and IE) as compared to the cheap wines (CC and IC). The labels of the expensive wines had two different designs. The label of the CE wine had a traditional label, while the IE had a rather modern label. Interestingly, participants were not aware of the difference in price range among the wines; thus, price did not influence their preferences. Similarly, the style of the label (modern vs. traditional) did not play a role. However, both labels clearly showed the production year, the name of the wine, and producer, and they had bronze and golden patters. Thus, participants may have preferred these labels due to the color and information provided on the labels (CE and IE), which may have increased the overall aesthetic appeal of the wine label. Our results suggest that different elements of the wine label may have influenced the perceived value and quality of the product. This confirms discussion in the literature that labels should offer reassurance that the wine offers value for money in terms of quality (Thomas and Pickering, 2003; Barber et al., 2006). Similar to the CE, the label of the IC wine displayed the type of grape variety (Cabernet Sauvignon), name of the wine, and the producer. As well as this, the IC wine had similar colors to the IE wine (red and goals). Consumer often gravitate toward brightly colored labels, such as gold, and other elements of the wine such as its appellation, country of origin, and year of production (Johnson and Bruwer, 2007; Hall and Mitchell, 2008; Terrien and Steichen, 2008; Atkin and Johnson, 2010; Famularo et al., 2010; Elliot and Barth, 2012; Larson, 2012). It is possible that some aspect of the IC label might have affected participant’s attention when they were initially exposed to the label. However, when participants had to make a conscious choice about the IC wine, they did not find the label aesthetical appealing. Consumers make a choice also based on what impression the label makes, in terms of content’s authenticity and quality. Thus, a comparison of the IC and CC labels with more expensive wines might have influenced participants’ preferences for it. This suggests that participants’ perceived quality for the IC label was less than for expensive wines (CE and IE).

The analysis of reaction times, recorded when participants classified the wine label, also confirms these findings. Research shows that the more we like something, the faster we tend to respond (Ramsøy, 2014; Calvert et al., 2019; Kim et al., 2020). Our results reveal that participants had shorter RT for the most preferred label CE. Vice versa, the CC label had the longest reaction time. Thus, the RT measure for CC is significantly higher than for the other three wines, confirming that this was the least preferred wine. It is interesting to notice that there was a slightly shorter mean response latency for the IE label compared to IC. On average, participants took the same amount of time to select IE and IC. Consumer neuroscience research shows that the closer in values two options are, the harder would be for a person to decide between them (Ramsøy, 2014; Kim et al., 2020). This might suggest that participants did not have a strong preference for one of the two labels, like for the CE label. Another possible explanation is that the characteristics of the IC label (design and bottle shape) might have influenced participant’s attention when they were initially exposed to the label. This is also confirmed by the consumer preferences and EEG data.

Several studies indicated that consumers’ preferences for a product are strongly influenced by visual attention (Glimcher and Fehr, 2013; Karmarkar and Plassmann, 2019). Consumers tend to choose the items that they look at initially or look at for the longest duration (Reutskaja et al., 2011; Laeng et al., 2016). We investigated whether the presence of the PCN could be associated with participant’ preference for a specific wine labeling. EEG data clearly show that the amplitude of the PCN differed between the employed wine labeling. The PCN clearly differed between the wine labeling from 80 to 240ms after stimulus presentation. In particular, the data suggest the presence of a PCN for two of the presented labels (CE and IC). The PCN was present for the CE and IC labels, which partially confirms the pattern present in a separate analysis of the behavioral data, as the most preferred label was CE.

Based on our interpretation, the findings suggest that the allocation of visual attention (reflected in the PCN) toward a product might reflect a preference for it but our results also show that this allocation may occur as specific features attract attention. Thus, the PCN component could be used in consumer neuroscience research to study how consumers’ visual attention mechanisms and linked to preferences. Overall, the findings of our study suggest that the final choice of a consumer and consequently their preference for a product could be influenced by both visual attention mechanisms and more complex cognitive processes where the outcome may be reflected in the activation of hand motor areas.^5^

## Conclusion

This study aims to bridge gaps in the consumer neuroscience literature on external product characteristics and in particular the effect of labels in product choice.

We contribute to previous research by studying the allocation of visual attention for product external characteristics. Several consumer neuroscience studies use eye tracking to examine visual attention mechanisms for wine labels. In our research, we used EEG to study whether different label design can influence both consumer preferences and brain activity.

By combining behavioral and EEG data, this study provides a more nuanced understanding of how neural activity related to visual attention can influence consumer information processing and decision-making. In addition, EEG research often focuses on brain waves or ERP components to investigate the attention-allocation behavior of consumer during product evaluation. To our knowledge, this is the first consumer neuroscience study that uses PCN components to assess visual attention mechanisms for wine labels.

We believe this study also has potential implications for companies and marketing practitioners. In particular, this study may serve as a reference for wine producers. The creative process behind designing a label for a particular wine has become increasingly complex and expensive, as wine companies are more frequently helped by professional designers specialized for the wine market. The findings of this study also suggest that increased attention for a product often but not always reflects a preference for it. Studying visual attention mechanisms helps companies to determine when (1) individual preference for a product is directly related to its visual saliency or is modulated by more high-level information and (2) product can catch consumers’ attention as well as influence their preferences, thus whether the consumer is attracted to the product, but he/she is also willing to buy it.

To conclude, the use of Consumer Neuroscience tools, such as EEG, can help to shed light on the cognitive and neuronal mechanisms that play a role during the exposure to product external characteristics. This might help companies to improve visual saliency tests by identifying whether specific product external characteristics have a strong impact on individual preferences.

### Limitations and Future Work

This study comes with some limitations that could be addressed in future research. In this study, we selected only four wine labels of the same type of grape to analyze consumers’ visual attention mechanisms and preferences. For future work, we suggest researchers use different types of wine (e.g., different grape variety). This will help recreate a real-life scenario in which participants choose from different wine types. As well as this, a higher number of labels might provide a more accurate analysis of which label characteristics influence consumers’ preferences (Ballester et al., 2005). We also believe that the experiment could be carried out using the method called A/B testing, whereby one half of the respondents (A) is shown the original version of the label and the second half (B) the modified label. This will help improve our understanding of how studying visual mechanisms can help companies improve their advertisement effectiveness, especially for e-commerce. This could also be applied to other type of products (e.g., detergents and clothing). Finally, designing an experiment well is important to improve the quality of Consumer Neuroscience research (Fink et al., 2007; Murray and Antonakis, 2019). In this study, our goal was to investigate whether changes in the brain activity and individual preferences for wine labels are related to the allocation of visual attention using PCN; hence, we consider this an exploratory study. We would like to invite researchers to replicate this study by further developing different aspects of the experiment procedures (e.g., using a prestudy to select the wine labels) or to collect data from different consumer neuroscience tools (simultaneously) to further improve our understanding of consumer preferences and visual attention mechanisms.

## Summary

We summarize the findings of our study as follows:

- The consumer neuroscience literature suggests that physiological and neurophysiological tools have been used to study whether visual attention mechanisms influence consumers’ preferences.
- The consumer neuroscience literature suggests that EEG has been used to investigate the influence of external product characteristics on participants’ preferences and brain activity.
- The current study shows that EEG provides relevant information about the allocation of visual attention.
- The current study shows that the PCN is an electrophysiological marker that can be used to examine the deployment of visuospatial attention for marketing stimuli.

## Data Availability Statement

The raw data supporting the conclusions of this article will be made available by the authors, without undue reservations.

## Ethics Statement

The studies involving human participants were reviewed and approved by The Ethical Committee of the BMS faculty, University of Twente. The patients/participants provided their written informed consent to participate in this studies.

## Author Contributions

All authors of this article had a significant contribution in its preparation. LA and RL created and designed the study and analyzed and interpreted the EEG data. LA collected the data. LA, RL, and EC reviewed and edited the manuscript. All authors contributed to the article and approved the submitted version.

## Conflict of Interest

The authors declare that the research was conducted in the absence of any commercial or financial relationships that could be construed as a potential conflict of interest.

## Publisher’s Note

All claims expressed in this article are solely those of the authors and do not necessarily represent those of their affiliated organizations, or those of the publisher, the editors and the reviewers. Any product that may be evaluated in this article, or claim that may be made by its manufacturer, is not guaranteed or endorsed by the publisher.

## Footnotes

^1^In the literature, we find different classifications of Neuromarketing and Consumer Neuroscience research (Lee et al., 2006; Hubert and Kenning, 2008; Plassmann et al., 2010; RamsÃ¸y, 2014). Consumer neuroscience research can be defined as the study of neuropsychological mechanisms that support and lead consumer decision making and behaviour (Alvino et al., 2020), while Neuromarketing is the application of neuroscientific methods for conducting company-specific market research (Hubert and Kenning, 2008). Thus, Consumer Neuroscience is considered as a scientific approach, while Neuromarketing is the application of Neuroscience methods to sell products.

^2^Other studies often use the term N2-posterior contralateral (N2pc); however, here we prefer the term PCN as the posterior contralateral negativity is not necessarily related to the N2 component.

^3^In this study, the wine labeling refers to both the front wine label design and bottle shape, as reported by Elliot and Barth (2012) and Laeng et al. (2016).

^4^The Greenhouse–Geisser correction was used for Îµ < 0.75, and the Huynh-Feldt correction was used for Îµ > 0.75.

^5^We verified whether individual differences in PCN amplitude for the CE label within the 200-240 and the 240-280 ms time intervals are predictive for individual preferences. Results revealed negative correlations for both time intervals (200-240 ms; r = -0.48, p = 0.007; 240-280 ms; r = -0.59, p = 0.001). These findings indicate that the more negative the individual PCN amplitude is for a specific label, the more likely it is that this participant will select this label.
